# Comprehensive Genomic Analysis of the Endophytic *Bacillus altitudinis* Strain GLB197, a Potential Biocontrol Agent of Grape Downy Mildew

**DOI:** 10.3389/fgene.2021.729603

**Published:** 2021-09-27

**Authors:** Qingchao Zeng, Jianbo Xie, Yan Li, Tantan Gao, Xun Zhang, Qi Wang

**Affiliations:** ^1^Department of Plant Pathology, MOA Key Lab of Pest Monitoring and Green Management, College of Plant Protection, China Agricultural University, Beijing, China; ^2^Beijing Advanced Innovation Center for Tree Breeding by Molecular Design, Beijing Forestry University, Beijing, China

**Keywords:** *Bacillus altitudinis*, endophyte, genome analysis, biocontrol, grape downy mildew

## Abstract

*Bacillus* has been extensively studied for agricultural application as a biocontrol agent. *B. altitudinis* GLB197, an endophytic bacterium isolated from grape leaves, exhibits distinctive inhibition to grape downy mildew based on unknown mechanisms. To determine the genetic traits involved in the mechanism of biocontrol and host-interaction traits, the genome sequence of GLB197 was obtained and further analyzed. The genome of *B. altitudinis* GLB197 consisted of one plasmid and a 3,733,835-bp circular chromosome with 41.56% G + C content, containing 3,770 protein-coding genes. Phylogenetic analysis of 17 *Bacillus* strains using the concatenated 1,226 single-copy core genes divided into different clusters was conducted. In addition, average nucleotide identity (ANI) values indicate that the current taxonomy of some *B. pumilus* group strains is incorrect. Comparative analysis of *B. altitudinis* GLB197 proteins with other *B. altitudinis* strains identified 3,157 core genes. Furthermore, we found that the pan-genome of *B. altitudinis* is open. The genome of *B. altitudinis* GLB197 contains one nonribosomal peptide synthetase (NRPS) gene cluster which was annotated as lichenysin. Interestingly, the cluster in *B. altitudinis* has two more genes than other *Bacillus* strains (*lgrD* and *lgrB*). The two genes were probably obtained *via* horizontal gene transfer (HGT) during the evolutionary process from *Brevibacillus*. Taken together, these observations enable the future application of *B. altitudinis* GLB197 as a biocontrol agent for control of grape downy mildew and promote our understanding of the beneficial interactions between *B. altitudinis* GLB197 and plants.

## Introduction

Grape is an ancient fruit crop which is also one of the most important in the modern world, based on its high economic value (Su et al., 2018). However, grape downy mildew, a widespread and destructive grapevine disease caused by *Plasmopara viticola*, reduces grapevine yield and wine quality (Zhang et al., 2017). This pathogen has a polycyclic behavior and infects all the green parts of the host leading to quantitative yield losses and qualitative damage (Gessler et al., 2011). Generally, to ensure the quantity and the quality of the harvest, the management of downy mildew requires a massive use of fungicides. However, overuse of the fungicides causes unintentional secondary effects on the environment and human health (Krzyzaniak et al., 2018). Therefore, several actors, including researchers and farmers, are now using alternative or complementary strategies, such as the use of biocontrol products, to control the disease (Gessler et al., 2011). Thus, biofertilizers and biopesticides may become the preferred substitutions for some conventional synthetic products.

Currently, biocontrol bacteria including the species of *Bacillus*, *Pseudomonas* and other genera of plant growth-promoting bacteria used in the prevention and management of plant diseases (Law et al., 2017). These species are widely known for their versatile metabolic activity and diverse beneficial effect on plant health. Moreover, their beneficial action can be expressed on a large range of plants which places these bacteria among the best candidates for the development of biopreparations. However, despite these positive characteristics, bacterial products can show some inconsistency between trials. This is assumed to be due to the short persistence of bacterial cells in the rhizosphere/soil environment and their susceptibility to unfavorable environmental conditions (Martin et al., 2015). One possible way to overcome these drawbacks is to develop bioproducts based on beneficial endophytic bacteria.

Plant-borne bacteria can live on the surface or inside their hosts, establishing bonds at different levels, ranging from a loose, free-living lifestyle in the vicinity of the host, to a tight association inside tissues (Little et al., 2008). An endophytic lifestyle benefits a microorganism by providing shelter, facilitating access to carbon sources, and increasing its overall fitness. In turn, endophytes can positively influence plant growth and its resistance to different stresses. A variety of plant growth promotion and biocontrol features related to nitrogen fixation, induction, or enhancement of plant defense mechanisms, synthesize molecules involved in plant protection against pathogens can be expected for endophytic bacteria (Hurek et al., 2002; Iniguez et al., 2005; Clarke et al., 2006; Bordiec et al., 2011). Since bacterial endophytes colonize the plant interior, which is a stable and protected environment, their interaction with plant can grow into a longer relationship. Such an ability can be exploited as a strategy to achieve a more sustainable and effective crop production system.

*Bacillus* genus which are ubiquitous in nature include an important number of species which produced a wide rand of secondary metabolites displaying inhibition to the disease. The spore-forming *Bacillus* used for biological preparations are preferred owing to their long-term viability that facilitates the development of products (Wu et al., 2015). Meantime, the *Bacillus* genus is also taxonomically diverse. The bacteria of some *Bacillus* groups usually share high genetic homogeneity including *B. cereus* and *B. subtilis* group (Liu et al., 2015; Chun et al., 2019). The sensibility of the method using 16S rDNA for strain classification is obviously reduced. It is reported that some isolates actually belong to the species of *B. altitudinis* rather than *B. pumilus* based on the housekeeping genes (Liu et al., 2013). With the development of sequencing, phylogeny based on the single-copy core genes has become a standard measure in the last several years (Xu et al., 2017). *B. altitudinis* is a bacterial species belonging to the *Bacillus* genus; several of them are of high ecological and biotechnological relevance. *B. altitudinis* is ubiquitous in the environment and has been isolated from plant (root and leaf), rhizosphere soil, soil, and animals (bull and buffalo dung) (Lu et al., 2017; Potshangbam et al., 2018; Thite and Nerurkar, 2020; Wang et al., 2021; Yue et al., 2021). Importantly, *B. altitudinis* has a high economic relevance owing to the wide range of applications that this microorganism and its products have in the industry, such as producing xylanolytic enzyme and the alleviation of Cu-induced phytotoxicity (Thite et al., 2020; Yue et al., 2021). Moreover, several *B. altitudinis* strains are also used as plant growth promoters. For example, the endophytic bacterium, *B. altitudinis* 19rs3 and T5S-T4, has been displaying growth promotion and *B. altitudinis* JSCX-1 has been used as a biocontrol agent against *Phytophthora sojae* (Lu et al., 2017).

In the present study, *B. altitudinis* GLB197 exhibited a strong antagonistic activity against various plant pathogens especially for the grape downy mildew in leaf disk and field experiments (Zhang et al., 2017). To gain a comprehensive understanding of the biocontrol potential of strain GLB197, we sequenced the genome of *B. altitudinis* GLB197 and conducted a phylogenetic analysis of GLB197 using 18 previously sequenced *B. pumilus* group strains. However, the ANI values between different *B. pumilus* group strains displayed quite striking differences. Furthermore, we found that the pan-genome of *B. altitudinis* is open. The lichenysin probably plays an important role in control the grape downy mildew. Importantly, we found that the lichenysin gene cluster in *B. altitudinis* GLB197 have two more genes than other *Bacillus* strains (*lgrD* and *lgrB*). The two genes probably were obtained via HGT during the evolutionary process from *Brevibacillus*. Moreover, *in silico* analyses allowed the identification of traits commonly associated with plant-bacteria interaction in GLB197. This work offers a foundation for follow-up studies of target genes and functions and facilitates a genetic engineering of *B. altitudinis* GLB197 to improve agricultural and industrial applications. This study also provides a scientific basis for the further optimization of the filed applications of the microbial biopesticide derived from *B. altitudinis* GLB197.

## Results

### Biocontrol Activity and Colonization of *Bacillus altitudinis* GLB197

**FIGURE 1:**
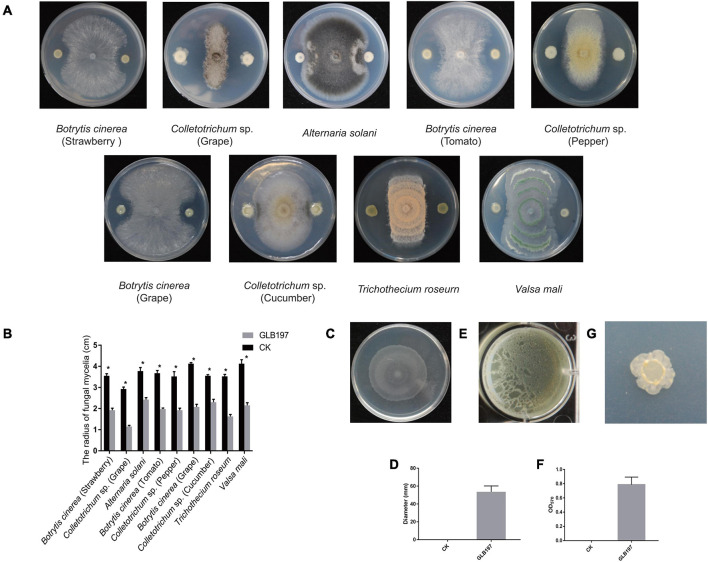
The features of *B. altitudinis* GLB197. **(A)** The antagonistic activity of GLB197 against nine pathogenic fungi on PDA plate. **(B)** Quantification of antagonistic activity of GLB197. *p < 0.01—significant differences compared with the control. **(C)** Swarming ability of GLB197 was assessed. **(D)** Data presented are the swarming ability of GLB197 on LB plates 5 h later. The data are expressed as the mean ± SD (*n* = 5). CK represented by LB medium and GLB197 represented by the strain. **(E)** Pellicle formation by GLB197 on Msgg liquid media. **(F)** OD readings from 12-well plate assays of biofilm formation by GLB197. The data are expressed as the mean ± SD (*n* = 6). **(G)** The colony morphology on MSgg plates was monitored for strain GLB197.

*Bacillus altitudinis* GLB197 was examined for its antagonistic potential against fungal pathogens. Results demonstrated that GLB197 significantly inhibited the mycelia growth of nine fungal pathogen with a clear inhibition zone (Figures 1A,B). The endophyte exhibited broad-spectrum antagonistic activities. To understand the cell motility and biofilm formation of the endophytic strain, the swarming motilities and biofilm formation of GLB197 was investigated. The GLB197 exhibited excellent swarming motility and colonized more than half the plate after 5 h of growth (Figures 1C,D). Meantime, GLB197 formed colonies composed of dense wrinkles and compact structures on a MSgg plate and formed robust, wrinkled pellicles on MSgg liquid medium (Figures 1E–G). During the period of 0–48 h, the level of cells accumulated in the biofilm increased about fivefold for GLB197. A previous study has shown that GLB197 could recolonize in grapevine leaves 5 weeks later (Zhang et al., 2017).

In summary, we have isolated *B. altitudinis* GLB197 from grape leaf, showing broad-spectrum antagonistic activities. Above all, the endophytic strain GLB197 is a beneficial microbe with potential for biotechnological application.

### General Genome Description of *Bacillus altitudinis* GLB197

The genome of *B. altitudinis* GLB197 consists of one chromosome and one plasmid encoding a total number of 3,781 coding DNA sequences (CDS). Among the predicted CDSs, 2,905 of them could be assigned a putative function, whereas 876 were predicted to encode hypothetical proteins. The total length of the chromosome genome was 3,733,835 bp, with a mean G + C content of 41.56%. This chromosome genome contained 3,770 CDS that made up 87.82% of genome. Furthermore, a total of 80 tRNA-coding genes and 24 rRNA genes were predicted in the chromosome sequence. Meanwhile, the GLB197 harbors a plasmid of 7,061 bp. The G + C content of the plasmid is 35.14% and lower than in the chromosome. The genomic features of *B. altitudinis* GLB197 are shown in Table 1 and Supplementary Figure 1.

**TABLE 1:** Summary statistics and information on the genome of the endophyte *Bacillus altitudinis* GLB197 in this study.

	Chromosome	Plasmid	Total
Genome size (bp)	37,33,835	7,061	37,40,896
G+C content	41.56%	35.14%	41.54%
Number of CDSs	3,770	11	3781
Coding percentage	87.82%	69.00%	87.78%
Average of ORF length	870 bp	443 bp	869 bp
rRNA	24	0	24
tmRNA	1	0	1
tRNA	80	0	80

### Whole-Genome Phylogenetic Analysis of *Bacillus altitudinis*

**FIGURE 2:**
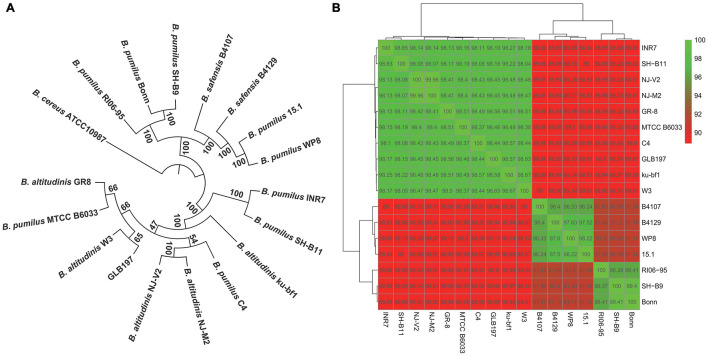
Phylogenetic tree of *Bacillus*. **(A)** Maximum likelihood tree of different *Bacillus* constructed based on 1,226 single-copy core proteins shared by 17 genomes and an outgroup (*Bacillus cereus* ATCC 10987). The phylogenetic tree was rooted by the outgroup. Support values of phylogenetic tree are shown for nodes as maximum likelihood bootstrap. **(B)** Heat map of average nucleotide identity values among different strains of *Bacillus* revealing three groups.

The phylogenetic tree of the 17 *Bacillus* genomes was constructed based on the concatenation of the 1,226 core genes that were present in single copy in all genomes with ML method and rooted by *B. cereus* ATCC 10987. It displayed that GLB197 and some *B. pumilus* and *B. altitudinis* strains formed a big cluster (Figure 2A). Meantime, the phylogenetic analysis revealed that GLB197 was a sister group to W3, which was isolated from raw gallnut honey in China. The sister group of GLB197 and W3 was a clade sister of strains GR-8 and MTCC B6033 (Figure 2A). However, the results showed that they are not distinguished by their geographical origin and the mixed trend of phylogenetic clustering of strains isolated from a similar environment. The results indicated that the phylogenetic clustering of genomes was apparently different from the habitat-specific grouping of these strains.

Furthermore, to confirm the findings from phylogenetic analysis, we also calculated the ANI values of different strains. The results confirmed that there are three major groups of selected *B. pumilus* group strains (Figure 2B). Strains with ANI values >95% are considered to be the same species (Richter and Rossello-Mora, 2009). As shown in Figure 2B, 17 *Bacillus* strains clustered three parts. The ANI values of three cluster strains are quite striking. Additional genomic features of GLB197 and 16 *B. pumilus* group genomes, such as the sequence similarity, was analyzed (Supplementary Figure 2). The analysis indicated a close genetic relatedness of the strains belonging to *B. pumilus* group and showed that most regions within their genomes were conserved.

### The Pan-Genome Features of *Bacillus altitudinis*

**FIGURE 3:**
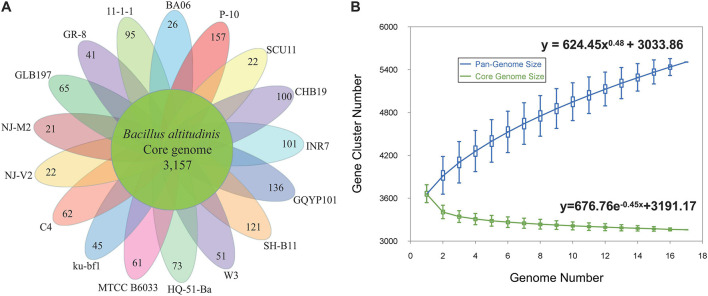
The pan-genomes of 17 *B. altitudinis* strains. **(A)** The number of unique CDS for each strain of the *B. altitudinis* pan-genome. The inner circle shows the core genomes shared between all strains. The specific genes for each strain are indicated in each of the outer circles. **(B)** Curves for *B. altitudinis* pan-genomes and core genomes. The blue dots denote the *B. altitudinis* pan-genome size for each genome comparison whereas the green dots indicate the *B. altitudinis* core genome size for each genome comparison. The median values were connected to represent the relationship between number of genomes and gene families.

A pan-genome for the strain GLB197 and 16 sequenced *B. altitudinis* strains was determined by the Pan-Genomes Analysis Pipeline (PGAP) software, comparing with the translated CDS set, followed by clustering of orthologous proteins and the representatives of each orthologous cluster and strain-specific CDS in the total pan-genome. The total pan-genome for the 17 compared *B. altitudinis* strains encompasses 5,506 CDS. Among the 5,506 protein-coding genes, 3,157 genes, which accounted for 57.34% of the genes in the pan-genome of *B. altitudinis*, were represented in all genomes. The accessory gene families (2,349 genes) were smaller than the core gene families (3,157 genes). The accessory gene families were further classified into 1,150 dispensable genes and 1,199 strain-specific gene families. The smallest numbers of specific genes were encoded by *B. altitudinis* strains NJ-M2 and NJ-V2 with 21 and 22, respectively. The highest numbers of unique genes (157) was found in *B. altitudinis* P-10 (Figure 3A).

The genome size for pan-genome and core genome among the selected *B. altitudinis* genomes was plotted against the number of genomes. The pan-genome curve displayed an asymptotic trend, indicating that 17 genomes were insufficient to describe the complete gene repertoire of the *B. altitudinis*. The generated pan-genome curves of *B. altitudinis* are well-represented by the Heaps law mathematical functions: *y* = 624.45*x*^0.48^ + 3033.86, where *y* refers to the pan-genome size while *x* refers to the number of sequenced genomes. According to these equations, the pan-genome size of *B. altitudinis* appeared to reach infinity when the number of genomes increase to infinity (Figure 3B). Therefore, our data suggest that *B. altitudinis* have open pan-genomes, which indicates that the species have infinite genomes. Analysis of the core genome was also asymptotic, with 3,157 core genes after the addition of the 17th genome. For these *B. altitudinis*, we estimated about 72 new genes detected when each additional genome is added from the mathematical equation (Supplementary Figure 3). The infinite pan-genome of *B. altitudinis* suggests the bacteria will keep acquiring new genes as they evolve independently over evolutionary time.

### Analysis of Genes Encoding Antifungal-Related Compounds

**FIGURE 4:**
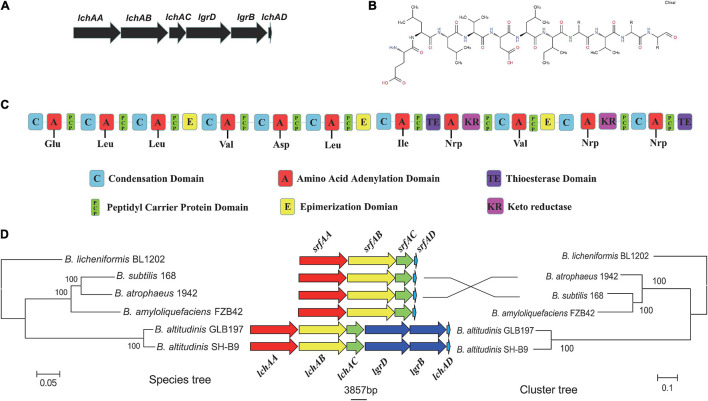
**(A)** The predicted biosynthetic gene cluster of lichenysin in GLB197. **(B)** The predicted structures of lichenysin in GLB197. **(C)** The schematic representation of the entire gene cluster for lichenysin and domain organization of synthetase gene coding for lichenysin in GLB197. **(D)** Maximum-likelihood (ML) phylogeny of single-copy core genes (1,889 core genes) and ML species phylogeny (six cluster genes) for the *Bacillus* strains.

**FIGURE 5:**
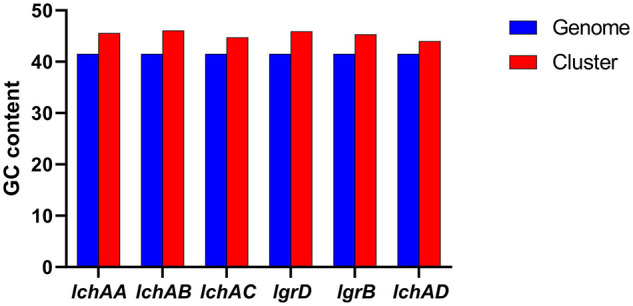
Comparison of G + C contents of the lichenysin clusters with the *B. altitudinis* GLB197 genomes.

It is noteworthy that the fermentation broth and cell-free supernatant of *B. altitudinis* GLB197 showed a distinctive inhibition to grapevine downy mildew on leaf disk and the sporangium release (data not published). Furthermore, the genome sequence of GLB197 was mined for the presence of gene-encoding lipopeptides. Only one gene cluster involved in nonribosomally synthesized lipopeptides was identified in the genome of GLB197 (Supplementary Table 1). The gene cluster probably plays an important role in antimicrobial activity. Figure 4A shows the six genes *lchAA*, *lchAB*, *lchAC*, *lgrD*, *lgrB*, and *lchAD* which are organized as a lichenysin gene cluster arranged within an 8.4-kb genomic region in *B. altitudinis* GLB197. Meantime, the putative product structure is displayed in Figure 4B. The modular organization of NRPSs involved in biosynthesis of lichenysin in *B. altitudinis* GLB197 may obey the linear rule (Figure 4C). Similarity searches against parts of publicly available *B. altitudinis* genomes revealed that the lichenysin gene cluster is also composed of the six genes (Figure 4D and Supplementary Figure 4). The result showed that the gene cluster is highly conserved among *B. altitudinis* strains. However, the G + C contents of the lichenysin cluster are higher than the genome of *B. altitudinis* GLB197 (Figure 5). Furthermore, we compared the gene cluster encoding lipopeptides of surfactin family between *B. altitudinis* and other *Bacillus* strains. We found that *Bacillus* strains such as *B. subtilis* and *B. atrophaeus* displayed that the gene cluster of surfactin family is composed of four genes (Figure 4D and Supplementary Figure 5). Importantly, the results raise the possibility that the two genes (*lgrD* and *lgrB*) were acquired through HGT from other strains. To test this hypothesis, individual phylogentic tree were reconstructed for all of the six cluster genes and their respective homologs identified across different bacterial genomes. The phylogenetic trees based on each of the individual *lchAA*, *lchAB*, *lchAC*, *lchAD*, *lgrD*, and *lgrB* protein sequences (Supplementary Figures 6–11) show that *lchABCD* of *Bacillus* are nested with those of *Paenibacillus*, implying that *Paenibacillus* and *Bacillus* have a common *lchABCD* gene ancestor. Whereas, the phylogeny show that *lgrB* and *lgrD* proteins of *B. altitudinis* fall into the *Brevibacillus* lineages, supporting that the ancestor of *lgrB* and *lgrD* may originate from *Brevibacillus*. Above all, it is most likely that the *lchABCD* cluster was transferred from *Paenibacillus* strains and then *B. altitudinis* strains acquired the *lgrD* and *lgrB via* HGT from *Brevibacillus*. However, the function and structure need to further confirm.

### Identification of Genes Involved in Plant Interaction

Endophyte uses multiple strategies to interact with their host. The “PIFAR” open-access web-based tool allowed the identification of several genetic factors involved in plant-bacteria interactions (Manuel Martinez-Garcia et al., 2016). A summary of the genes identified in *B. altitudinis* GLB197 is provided in Table 2. Specific information about genetic factors can be found in Supplementary Table 2. A set of known mechanisms, which endophytes often use to interact with plants, are found in the genome of *B. altitudinis* GLB197 such as chemotaxis (*cheA*, *cheW*, and *cheY*), EPs (*gpsX* and *galU*), plant adaptation and protection (i.e., four genes for siderophores, one genes for plant hormones, and 11 genes for detoxification), adhesion and biofilm (i.e., *attC* and six genes for biofilm) (Supplementary Table 2). For instance, the EPS is necessary for rhizoplane and endosphere colonization of rice plants in *Gluconacetobacter diazotrophicus* (Meneses et al., 2011). The genome analysis revealed genes which might be important for plant-bacteria interaction.

**TABLE 2:** Summary of genes identified in the genomes of *Bacillus altitudinis* GLB197 involved in plant-bacteria interaction.

Genetic factors involved inplant-bacteria interaction	Adhesion	Antibiotic	Biofilm	Detoxification	EPSs	MAMPs	MDRs	PCWDEs	Proteases	Volatiles	Siderophores	Metabolism	LPSs	Hormon es
*B. altitudinis* GLB197	1	25	6	11	2	16	12	5	1	4	4	10	1	z

## Discussion

Pathogenic microorganisms affecting plant health are a major and chronic threat to food production and ecosystem stability worldwide (Compant et al., 2005). Based on the adverse effects of some chemicals on human health, the environment, and living organisms, there is now a strong push to develop low-input and more sustainable agricultural practices that include alternatives to chemicals for controlling pests and diseases (Ab Rahman et al., 2018). There is a growing body of evidence that demonstrates the potential of microbiomes to increase plant efficiency and yield. However, their application as biofertilizers and biopesticides whose success in the field is still inconsistent. Martin et al., 2015 reported that environmental conditions could affect the efficacy of preventive treatments with endophytes under field conditions, despite the fact that these endophytes showed strong *in vitro* antagonism against the vascular pathogen (Martin et al., 2015). Importantly, the knowledge about basic mechanisms of interactions between bacilli and plants is insufficient. Several species of bacteria, such as, *Bacillus*, *Streptomyces*, and *Pseudomonas*, have been marketed as biological control agents (Paterson et al., 2017). However, biological preparations from spore-forming *Bacillus* sp. are preferred, because their long-term viability facilitates the development of commercial products. In this study, the isolate GLB197 displayed broad-spectrum antagonistic activities *in vitro*. Meantime, GLB197 exhibited robust preventive effects against grape downy mildew filed during two successive years (Zhang et al., 2017). The endophytic strain is a beneficial microbe with a potential for biotechnological application in the future.

The increasing sequenced microbial genomes provide an ideal opportunity to re-evaluate approaches in understanding phylogeny among bacteria (Alcaraz et al., 2010). Much of the understanding of microbial biodiversity has been studied by comparison of rRNA sequences. However, this approach has clear limitations, such as the inability to resolve relationships between closely related groups. In our analysis, we used 1,226 single-copy core genes to obtain a robust phylogenetic analysis. In the result, the tree divided into different clusters (Figure 2A). Furthermore, the heat map based on ANI values displayed an obvious difference. Interestingly, the ANI values divided the 17 strains into three groups (Figure 2B). The ANI values of the intragroup was higher (>96%). Nevertheless, the ANI values between intergroup was lower (<96%). Strains with ANI values >95% are considered to be the same species (Richter and Rossello-Mora, 2009). Some reported that the different lineages and sublineages representatives of *Staphylococcus epidermidis* show high ANI values (>96%). As a result, the taxonomy of some *B. pumilus* group strains should be queried (Figure 2B). Whole-genome sequencing (WGS) launched microbial taxonomy into the new era of genomic microbial taxonomy. The genomic taxonomy is to extract taxonomic information from WGS that can be used to establish a solid framework for the identification and classification of species (Thompson et al., 2013). Then, we should select more *Bacillus*-type strains to identify the taxonomic position of these *B. pumilus* group strains or reclassify the current position of *B. pumilus* group strains.

The genus *Bacillus* exhibits extensive environmental adaptability and can populate various ecological niches. Firstly, we investigated the pan-genome of the species *B. altitudinis*. The results show that the pan-genome of the *B. altitudinis* is open and theoretically infinite, suggesting that *B. altitudinis* species tend to acquire new genes to enhance the adaptability (Figure 3). Bacteria must change their genetic material to adapt to variable environmental conditions, thus, greater niche diversity reflects larger pan-genomes (Konstantinidis and Tiedje, 2004). Considering the wide distribution of *Bacillus*, a large pan-genome size corresponds to diverse living conditions. Meantime, pan-genome studies have also opened a new window to closely observed genetic determinants of endophytism. Pan-genome studies may therefore lead to the identification of signature genes responsible for adaption and evolution of a microbe as an endophyte (Kaul et al., 2016). The pan-genome analysis found that 65 unique to *B. altitudinis* GLB197. The unique genes of GLB197 need to further study. In general, the microbe is equipped with the essentials for the life styles, so there must be some external factors that make the microbe become an endophyte strain. Previous documents reported that the endosphere isolate has a stronger plant colonization ability than the soil isolate. Importantly, the global transcriptome profiles evidenced that the endophyte responded more pronounced than the soil-derived isolate to potato-root exudates (Yi et al., 2017). Based on the background, comparison of genomes of species inhibiting different habitats (endophyte and nonendophyte strains) is quite useful in disclosing the molecular determinants responsible for the distinction.

The main approaches of gain include HGT and gene diversification, and the former is more widespread in bacteria (Treangen and Rocha, 2011). The methods used to detect HGT are primarily divided into two categories: parametric and phylogenetic methods (Ravenhall et al., 2015). In the present study, the HGT region in *B. altitudinis* GLB197 was confirmed using various methods. The G + C contents of the lichenysin cluster are higher than the genome of GLB197 (Figure 5). According to the phylogenetic tree, *lchABCD* of *Bacillus* are nested with those of *Paenibacillus*, while *lgrB* and *lgrD* proteins of *B. altitudinis* fall into the *Brevibacillus* lineages (Supplementary Figures 6–11). Therefore, we suggested the following hypothetical HGT pathways for the gene clusters: first, one common ancestor probably *Paenibacillus* provided this cluster inserted into *B. altitudinis* and *Bacillus* strains; second, the *B. altitudinis* strains acquired two other genes (*lgrB* and *lgrD*) through HGT from *Brevibacillus* strains. Finally, we found that some genes are related to plant-bacteria interaction. However, as to who the donor is or where the origin of these genetic traits related to plant-bacteria interaction is still remains a puzzle. We should attempt to solve this problem in a further study.

## Materials and Methods

### Bacterial Strains and Growth Conditions

The *B. altitudinis* GLB197 used in this study, which has been described previously, was isolated from grapevine leaves (Zhang et al., 2017). GLB197 was routinely grown at 37°C on Luria-Bertani (LB) broth or on solid LB medium supplemented with 1.5% agar. For assays of biofilm formation, MSgg medium was used. The recipe for MSgg is as follows: 5 mM potassium phosphate (pH 7.0), 100 mM morpholine propane sulfonic acid (MOPS) (pH 7.0), 2 mM MgCl_2_, 700 mM CaCl_2_, 50 mM MnCl_2_, 50 mM FeCl_3_, 1 mM ZnCl_2_, 2 mM thiamine, 0.5% glycerol, 0.5% glutamic acid, 50 mg/ml tryptophan, 50 mg/ml threonine, and 50 mg/ml phenylalanine (Branda et al., 2001).

### *In vitro* Antifungal Activity

We performed a plate confrontation assay to test the activity of GLB197 against common fungal pathogens including *Colletotrichum* sp., *Alternaria solani*, *Botrytis cinerea*, *Valsa mali*, and *Trichothecium roseum*. In the plate confrontation assay, the fungi were cultivated on potato-dextrose-agar plates (PDA) at 28°C for 3–5 days. A 5-mm-diameter block of mycelium culture was cut and transferred into the center of a fresh PDA plates. After 1 day of incubation, 2 μl of the overnight cultures grown in LB medium was spotted on the PDA plate 2.5 cm away from the center, where the mycelium agar block was placed. The antifungal activity was evaluated by measuring diameter of mycelium after 5–7 days of incubation at 28°C. All of the plant pathogens are stored in the lab.

### Biofilm Formation and Swarming Motility Assay

In brief, 5 ml LB liquid cultures was prepared with shaking (200 rpm) at 37°C to OD_600_ = 0.8, 1 ml of cells were collected by centrifugation at 6,000 × *g* for 5 min, washed with phosphate-buffered saline (PBS, 137 mM NaCl, 2.7 mM KCl, 10 mM Na_2_HPO_4_, and 2 mM KH_2_PO_4_), and resuspended in 100 μl PBS. Swarming motility of GLB197 was tested using standard protocols with minor modifications. LB plates containing 0.7% agar were dried in a laminar flow hood for 20 min and then 3 μL of the cell suspension was spotted on the center of each plate (Chen et al., 2012). The plates are incubated at room temperature for 5 h to allow cell growth in order to clearly visualize the swarming zone. Then, the swarming agar plates were dried for another 2 h in a laminar flow hood. The diameter of the swarming zone was measured. The assay was performed with three independent experiments, each with at least three technical replicates.

To monitor pellicle formation, the biofilm formation was analyzed in MSgg medium. GLB197 was grown in LB medium at 37°C overnight. Then, 4 μl of culture was added to 4 ml of MSgg medium in 12-well plates and incubated statically at 28°C for up to 48 h. To quantify biofilm formation, the culture beneath the biofilm was drawn off carefully and the biofilm in each well was washed with 3 ml sterile saline three times and fixed with 2 ml of 99% (*v*/*v*) methanol for 15 min, followed by air-drying. The dried biofilms were stained with 2 ml 1% crystal violet (CV) for 10 min. Excess CV was then removed. The CV bound to the cells was dissolved in 5 ml of 33% (*v*/*v*) glacial acetic acid and diluted 200 times for OD_570_ detection (Weng et al., 2013). The assay was performed with three independent experiments.

### Genome Sequencing, Assembly, and Annotation

DNA concentration and purity were checked by a spectrophotometer (Nanodrop 2000, Thermo Scientific, Waltham, MA, United States), and the DNA integrity was analyzed by agarose gel electrophoresis. Then, the genomic DNA was sent to Tianjin Biochip Corporation (Tianjin, China), a commercial NGS service provider, for whole genome sequencing. The complete genome of GLB197 was sequenced by the Illumina HiSeq and Pacific Biosciences (PacBio, Menlo Park, CA, United States) platforms. Briefly, genomic DNA sample were fragmented using an ultrasonication approach (Covaris, Woburn, MA, United States) according to the manufacturer’s instruction. The 10-kb template library was constructed using DNA Template Prep Kit 2.0 with the 10-kb insert library protocol. The library was sequenced using the PacBio RSII Sequencer. Meantime, 1 μg DNA sample was also fragmented using an ultrasonication approach, size selected and end repaired. Each generated fragment was ligated to Illumina-specific adapter sequence, quantified, indexed, and sequenced on the Hiseq platform of Illumina. Quality-of-sequence reads were first analyzed using FastQC tool. Then, adaptor sequence removal, trimming, error correction, and assembly were performed using the HGAP software (Chin et al., 2013). Gene predictions were performed with Prokka version 1.11 which predicts coding DNA sequence (CDS) using Prodigal (Seemann, 2014). Annotation of the protein-coding sequence was conducted using the Basic Local Alignment Search Tool (BLAST) against the COG, Kyoto Encyclopedia of Genes and Genomes, and Interpro databases. The final annotated chromosome was plotted using CIRCOS to show the gene locations, GC skew, and GC content (Krzywinski et al., 2009).

### Phylogenetic Tree

We constructed a phylogenetic tree for *B. altitudinis* GLB197 and other 16 *Bacillus* genomes, and *B. cereus* ATCC 10987 was included as an outgroup. All sequences of *Bacillus* were downloaded from the National Center for Biotechnology information. A phylogenetic tree for the set of 18 genomes was inferred using a core-genome alignment concatenation approach. Multiple alignments of amino acid sequences were conducted using MAFFT (version 7.310) (Katoh and Standley, 2013), and conserved blocks from multiple alignments of test protein were selected by using Gblocks (Castresana, 2000). The maximum likelihood tree was constructed using the RAxML (version 8.2.10) software and the PROTGAMMALGX model with 100 bootstrap replicates. To evaluate the phylogeny of the *B. altitudinis* strains, ANI was calculated using the JSpecies software with MUMmer (NUCmer) alignment (Richter and Rossello-Mora, 2009). The phylogenetic tree was generated using MEGA (Kumar et al., 2008). Then, the heat map was visualized using R package as a confirmation. Moreover, pairwise genome alignment of all 16 *B. altitudinis* strains was performed and represented by BRIG 0.95. Single gene alignments were aligned with molecular evolutionary genetics analysis (MEGA). The neighbor-joining trees were constructed using the same software, and 500 bootstraps were done (Kumar et al., 2008).

### Pan-Genome Analysis

To identify the core and strain-specific genes, a pan-genome analysis of *B. altitudinis* strains was carried out by the PGAP software which implements functional ortholog clustering using the amino acid sequences based on Gene Family (GF) method (Zhao et al., 2012). PGAP pipeline-based protein similarity method was used to detect a set of core orthologs from the 17 *B. altitudinis* strains, and the core orthologs were clustered with at least 50% protein sequence identity to each other and 50% overlap with the longest sequence with an *e*-value 1*e*−5. The dataset of shared genes among the 17 strains was defined as their core genomes, and the total set of genes within test genomes was defined as the pan-genome. Strain-specific genes were extracted from the orthologous table by using Perl script.

The core and pan-genomes, as well as estimated respective sizes and trajectories were made using models and regression algorithms proposed by Tettelin et al. (2005, 2008). The curve fitting of the pan-genome was performed using a power-law regression model based on Heaps law [*y* = *Apanx*^*Bpan*^ + *Cpan*] as previously described (Tettelin et al., 2005, 2008), where *y* denotes pan-genome size, *x* the genome number, and *A*_*pan*_, *B*_*pan*_, and *C*_*pan*_ are fitting parameters. Here, *B*_*pan*_ is equivalent to the parameter γ used by Tettelin et al. (2005, 2008) in estimating the open or closed nature of a pan-genome (Rasko et al., 2008). When 0 < *B*_*pan*_ < 1, the size of the pan-genome increases unboundedly with sequential addition of new genomes and can be considered open. Conversely, when *B*_*pan*_ < 0 or > 1, the pan-genome trajectory approaches a plateau as further genomes are added and can be considered closed. The curve fitting of core genome was performed using an exponential regression model [*y* = *A*_core_*e*^(*B*_*core*_*x*)^ + *C*_*core*_] (Tettelin et al., 2005, 2008; Rasko et al., 2008). New gene plots were derived from the pan-genome showing the number of new “strain-specific” genes contributing to the pan-genome per additional sequenced strain as a function of the number of strains. Both the core and pan-genome were visualized through PanGP v1.0.1 software (Zhao et al., 2014). PanGP was run using default parameters generating distribution plots of (i) total genes, (ii) conserved genes, and (iii) new genes found upon progressive sampling of “*n*” genomes.

### Detection of Genetic Traits Involved in Secondary Metabolites Cluster and Bacteria-Plant Interaction

Secondary metabolite clustering was predicted using the antiSMASH website (Weber et al., 2015). Moreover, bioinformatics identification of genetic factors (i.e., adhesion, antibiotics, biofilm, detoxification, microbe-associated molecular patterns (MAMPs), multidrug resistance (MDRs), plant cell wall-degrading enzymes (PCWDEs), bacterial lipopolysaccharides (LPSs), siderophores, proteases, etc.) involved in plant-bacteria interaction was performed by implementing the PIFAR open-access, web-based tool (Manuel Martinez-Garcia et al., 2016).

### Nucleotide Sequence Accession Number

This whole genome shotgun project of *B. altitudinis* GLB197 has been deposited at DDBJ/ENA/GenBank under the accession CP018574.1.

## Data Availability Statement

The datasets presented in this study can be found in online repositories. The names of the repository/repositories and accession number(s) can be found in the article/Supplementary Material.

## Author Contributions

QZ, JX, and QW conceived the study and prepared the main manuscript. QZ, JX, YL, and TG carried out data analyses. QZ, JX, and XZ drafted the tables and figures. All authors participated in the experimental design and reviewed the manuscript.

## Conflict of Interest

The authors declare that the research was conducted in the absence of any commercial or financial relationships that could be construed as a potential conflict of interest.

## Publisher’s Note

All claims expressed in this article are solely those of the authors and do not necessarily represent those of their affiliated organizations, or those of the publisher, the editors and the reviewers. Any product that may be evaluated in this article, or claim that may be made by its manufacturer, is not guaranteed or endorsed by the publisher.

## Supplementary Material

Supplementary Figure 1 **(A)** Circular representation and subsystem category distribution of the chromosome of *B. altitudinis* GLB197. **(B)** Circular representation and subsystem category distribution of the plasmid of *B. altitudinis* GLB197. Each ring of the circle has a different genome information: rings from the outside in: (1) scale marks (unit: Mb), (2 and 3) protein-coding genes on the forward and reverse strand, respectively (color coded by the functional categories), (4 and 5) rRNA (blue) and tRNA (red) on the forward and reverse strand, respectively, (6) GC content (positive: red; negative: blue), and (7) GC skew (above average: aquamarine; below average: orange).

Supplementary Figure 2 Genome comparisons of other *B. pumilus* group strains against reference genome (strain GLB197) generated by BRIG 0.95; the circular map illustrates the whole genome comparison of strain GLB197 against the other 16 sequenced *B. pumilus* group strains. The inner cycle (back) represents the complete genome of the reference strain GLB197, and the shade of each colors show the similarities between each strains with strain GLB197.

Supplementary Figure 3 Curves for *B. altitudinis* illustrate the number of expected new genes detected with every increase in the number of *B. altitudinis* genomes.

Supplementary Figure 4 Organization of genes in *B. altitudinis* strains. The *lchAA*, *lchAB*, *lchAC*, *lgrB*, *lgrD*, and *lchAD* are marked with different colors.

Supplementary Figure 5 Organization of surfactin family genes in *Bacillus* strains. The different genes are marked with different colors.

Supplementary Figure 6 Neighbor-joining phylogenetic tree of the *lchAA* protein sequences derived from *Bacillus* and other representative species. A total of 500 bootstrap replicates were made, and bootstrap values are indicated at each node.

Supplementary Figure 7 Neighbor-joining phylogenetic tree of the *lchAB* protein sequences derived from *Bacillus* and other representative species. A total of 500 bootstrap replicates were made, and bootstrap values are indicated at each node.

Supplementary Figure 8 Neighbor-joining phylogenetic tree of the *lchAC* protein sequences derived from *Bacillus* and other representative species. A total of 500 bootstrap replicates were made, and bootstrap values are indicated at each node.

Supplementary Figure 9 Neighbor-joining phylogenetic tree of the *lchAD* protein sequences derived from *Bacillus* and other representative species. A total of 500 bootstrap replicates were made, and bootstrap values are indicated at each node.

Supplementary Figure 10 Neighbor joining phylogenetic tree of the *lgrB* protein sequences derived from *Bacillus* and other representative species. A total of 500 bootstrap replicates were made, and bootstrap values are indicated at each node.

Supplementary Figure 11 Neighbor joining phylogenetic tree of the *lgrD* protein sequences derived from *Bacillus* and other representative species. A total of 500 bootstrap replicates were made, and bootstrap values are indicated at each node.

Supplementary Table 1 Genes and gene cluster encoding for secondary metabolites in *B. altitudinis* GLB197.

Supplementary Table 2 Genes identified in the genomes of *B. altitudinis* strains GLB197 involved in plant-bacteria interaction according to the wed-based tool PIFAR.
