# Supplementary figures and images for: Comprehensive Genomic Analysis of the Endophytic Bacillus altitudinis Strain GLB197, a Potential Biocontrol Agent of Grape Downy Mildew
Source: Front Genet. 2021 Sep 27;12:729603. doi: 10.3389/fgene.2021.729603 (PMC8502975; doi:10.3389/fgene.2021.729603)

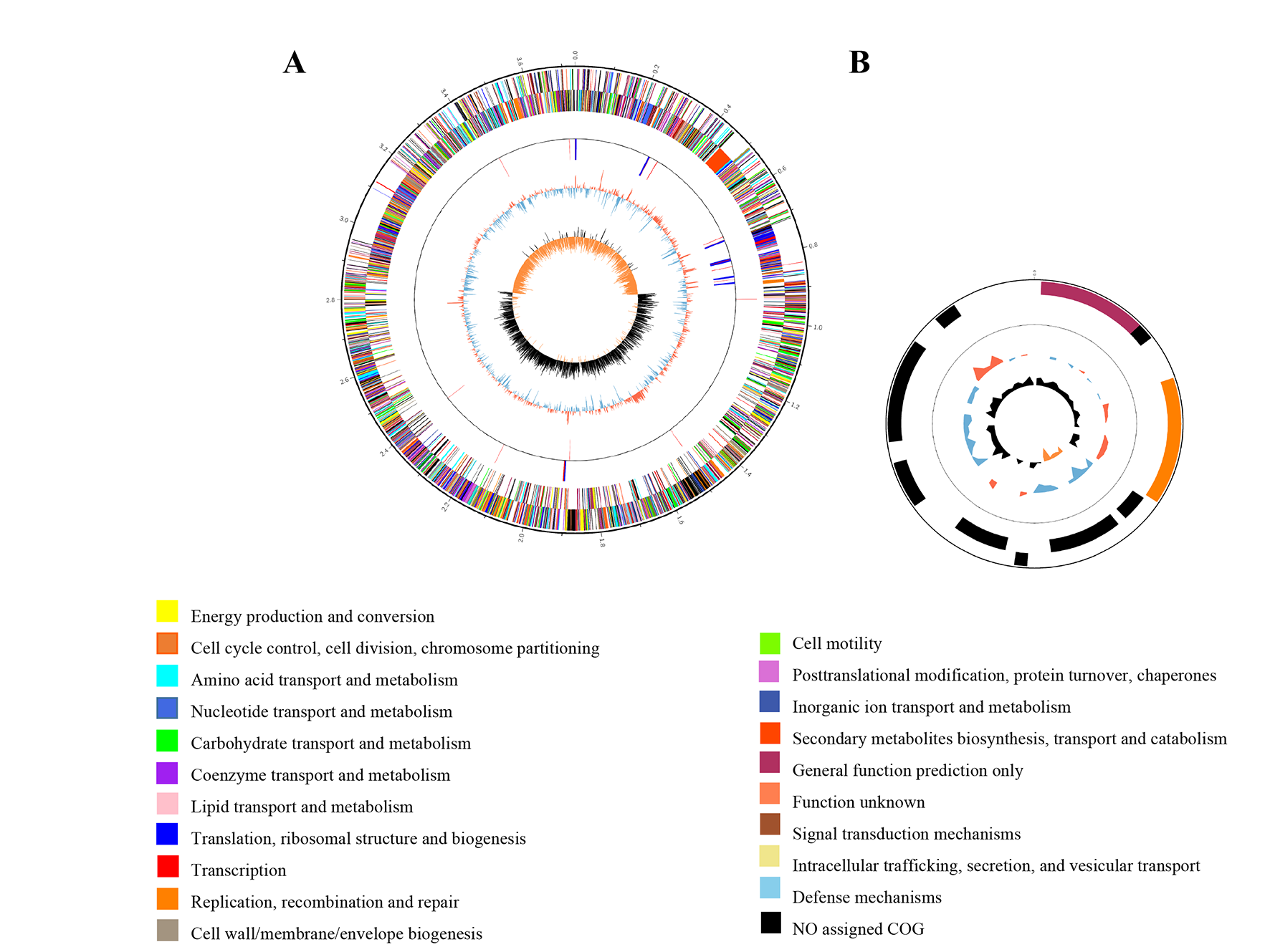

Supplement: Supplementary Figure 1 — (A) Circular representation and subsystem category distribution of the chromosome of B. altitudinis GLB197. (B) Circular representation and subsystem category distribution of the plasmid of B. altitudinis GLB197. Each ring of the circle has a different genome information: rings from the outside in: (1) scale marks (unit: Mb), (2 and 3) protein-coding genes on the forward and reverse strand, respectively (color coded by the functional categories), (4 and 5) rRNA (blue) and tRNA (red) on the forward and reverse strand, respectively, (6) GC content (positive: red; negative: blue), and (7) GC skew (above average: aquamarine; below average: orange). [file Image_1.TIF]

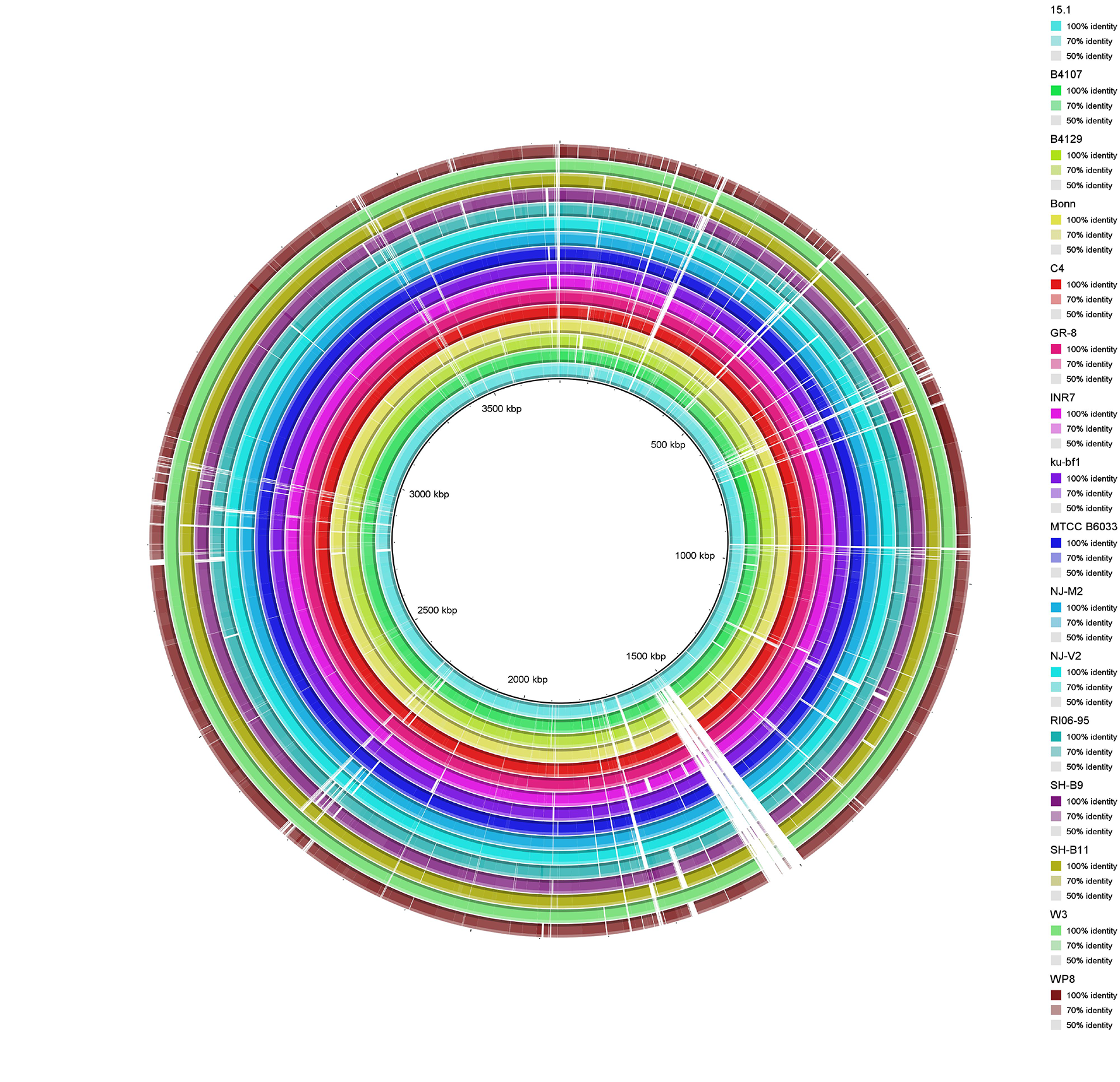

Supplement: Supplementary Figure 2 — Genome comparisons of other B. pumilus group strains against reference genome (strain GLB197) generated by BRIG 0.95; the circular map illustrates the whole genome comparison of strain GLB197 against the other 16 sequenced B. pumilus group strains. The inner cycle (back) represents the complete genome of the reference strain GLB197, and the shade of each colors show the similarities between each strains with strain GLB197. [file Image_2.TIF]

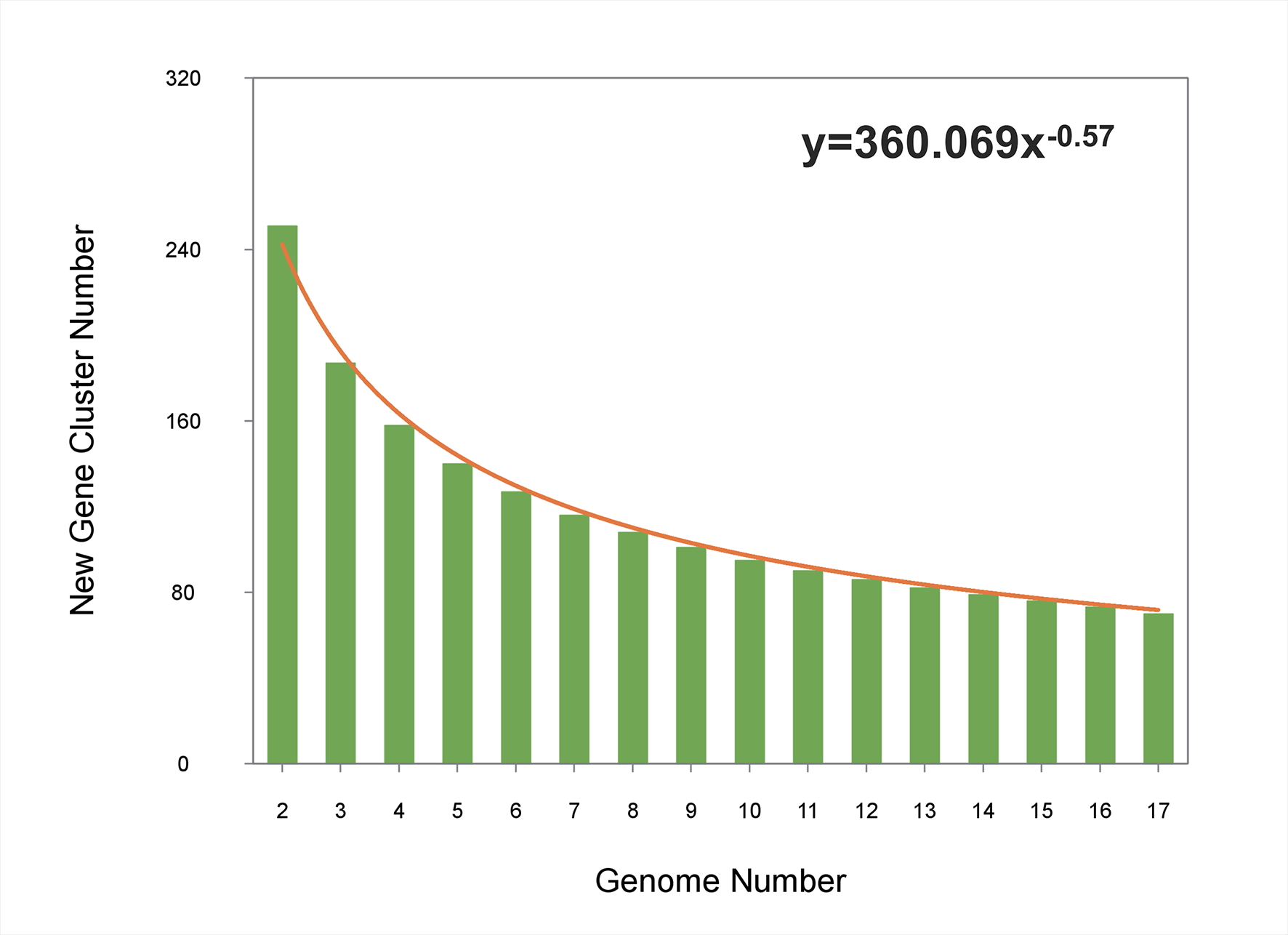

Supplement: Supplementary Figure 3 — Curves for B. altitudinis illustrate the number of expected new genes detected with every increase in the number of B. altitudinis genomes. [file Image_3.TIF]

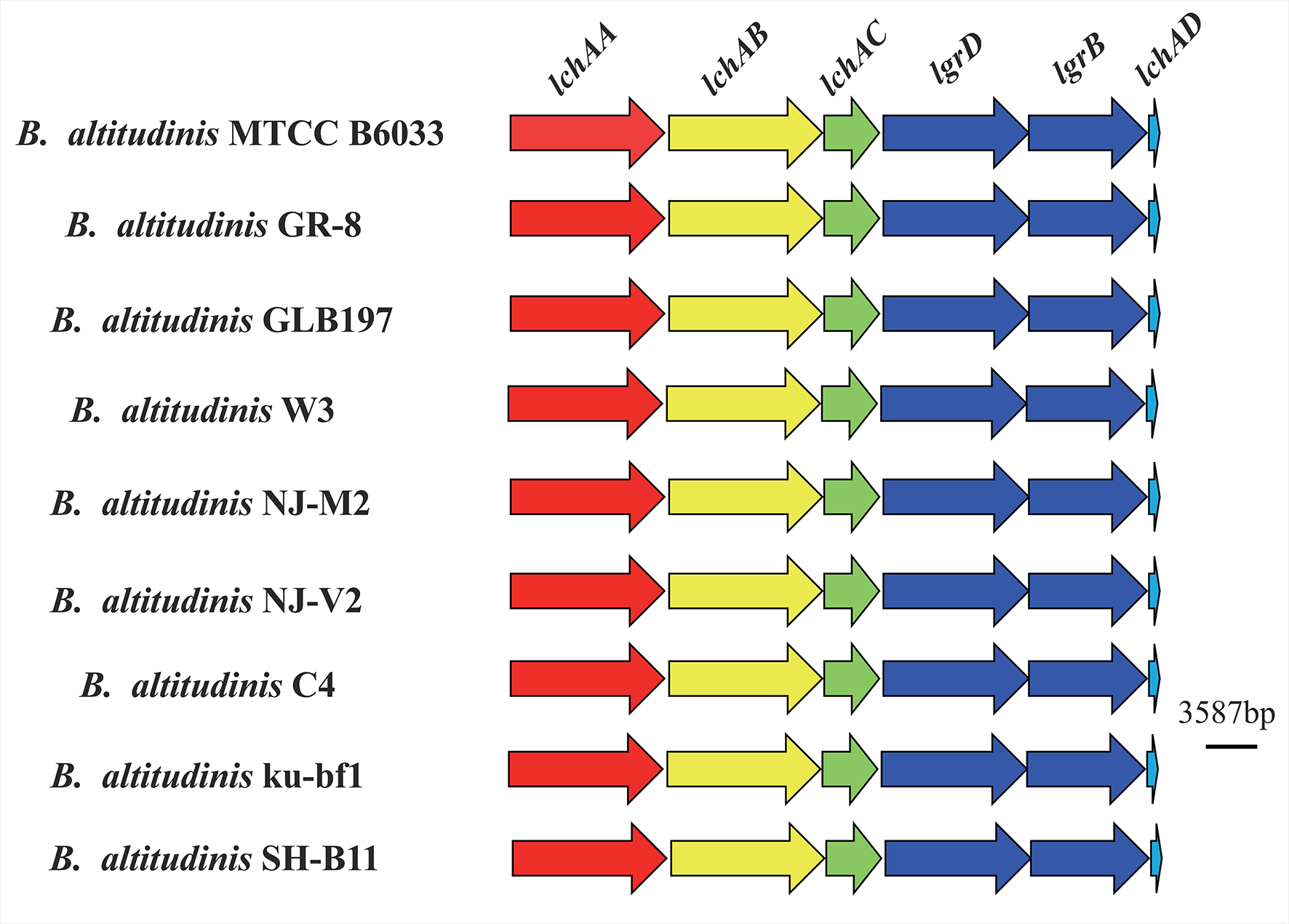

Supplement: Supplementary Figure 4 — Organization of genes in B. altitudinis strains. The lchAA, lchAB, lchAC, lgrB, lgrD, and lchAD are marked with different colors. [file Image_4.TIF]

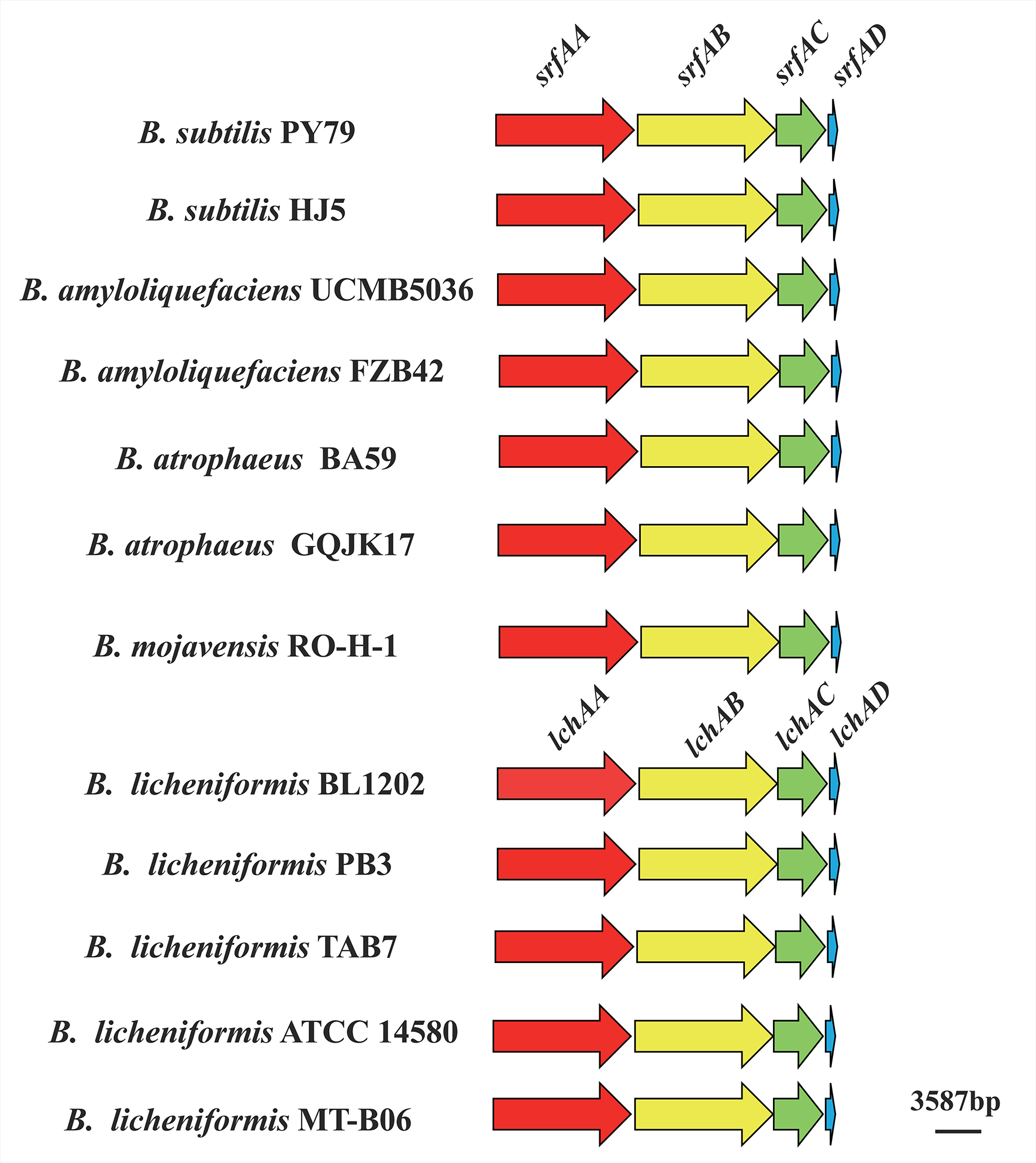

Supplement: Supplementary Figure 5 — Organization of surfactin family genes in Bacillus strains. The different genes are marked with different colors. [file Image_5.TIF]

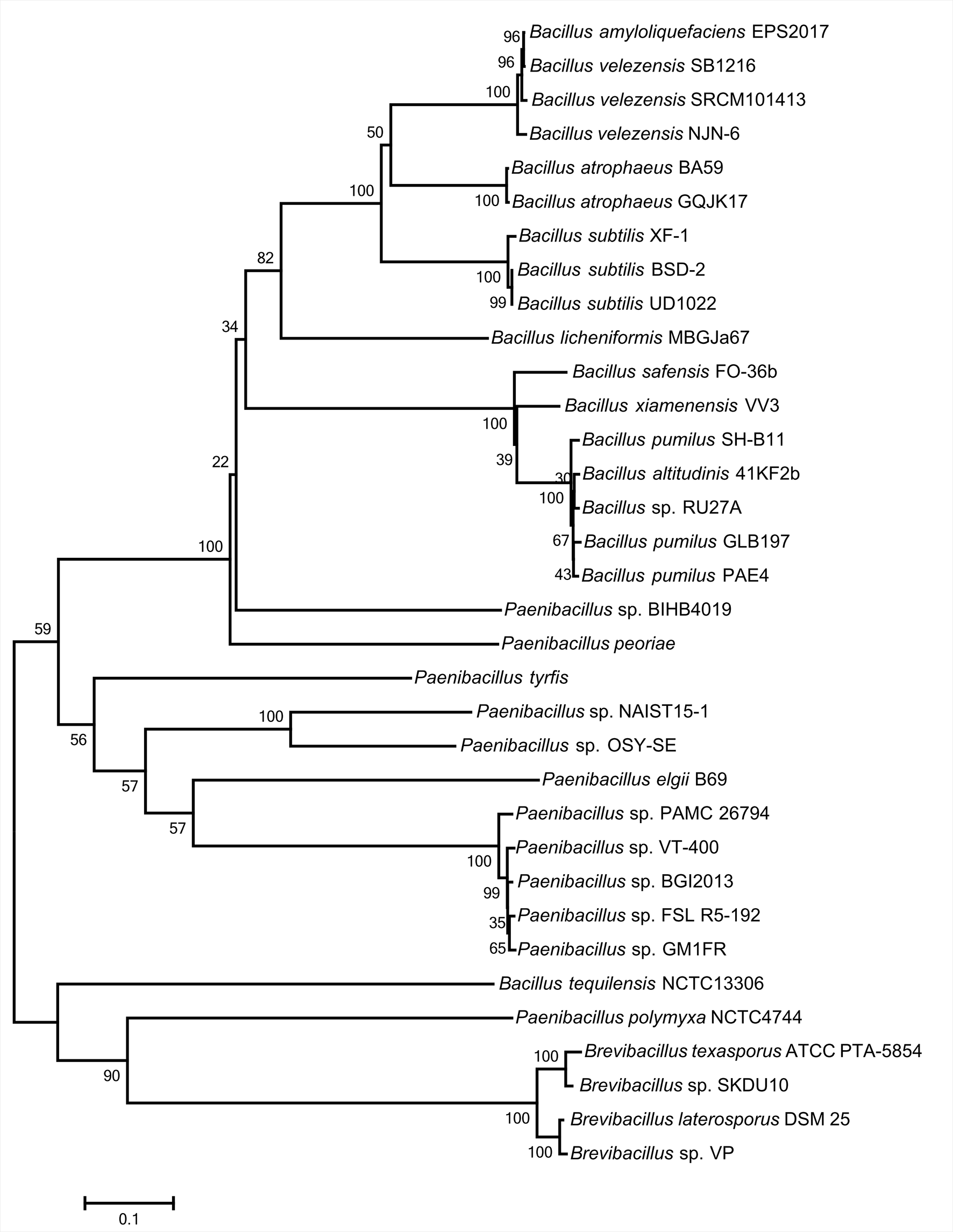

Supplement: Supplementary Figure 6 — Neighbor-joining phylogenetic tree of the lchAA protein sequences derived from Bacillus and other representative species. A total of 500 bootstrap replicates were made, and bootstrap values are indicated at each node. [file Image_6.TIF]

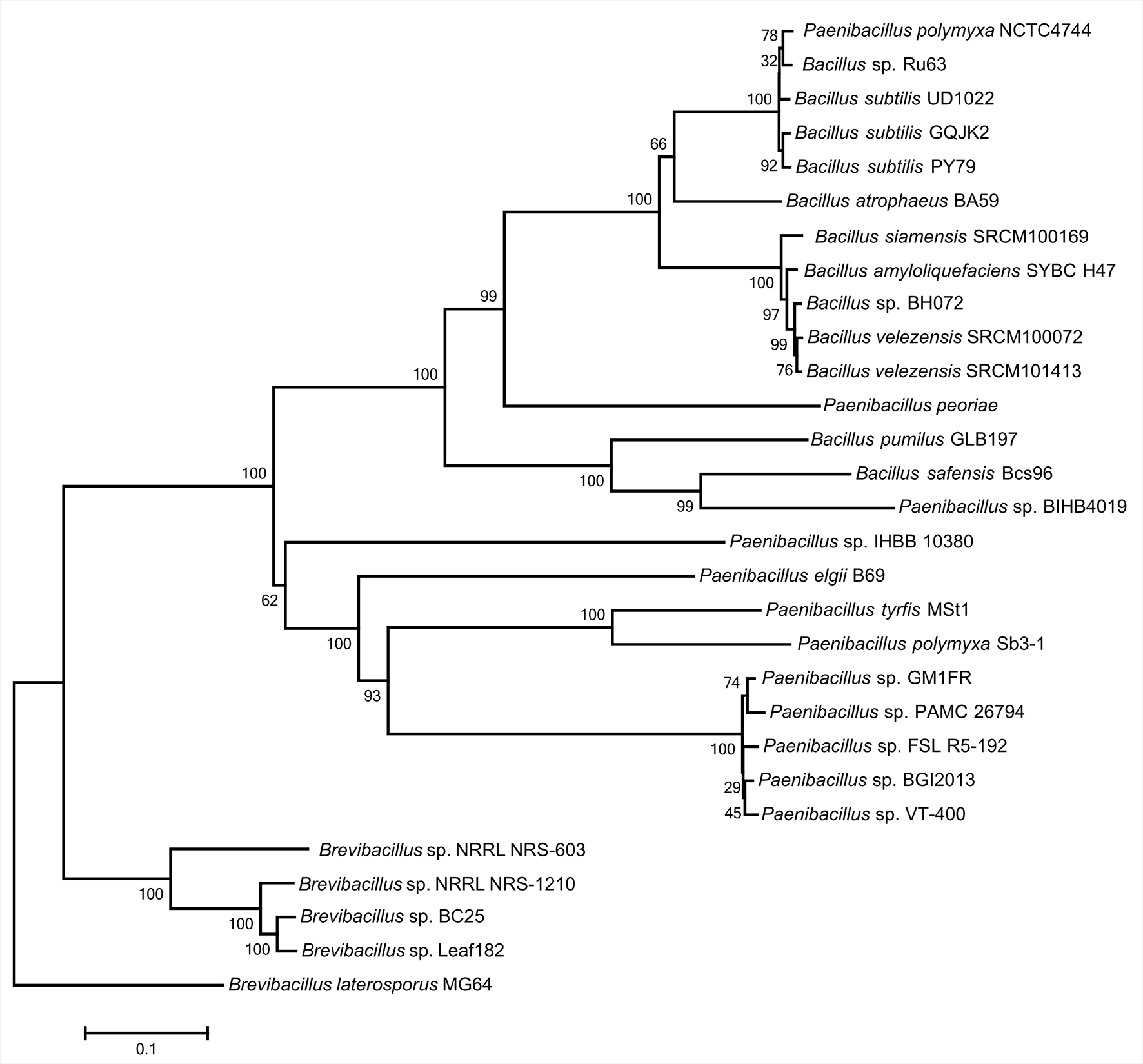

Supplement: Supplementary Figure 7 — Neighbor-joining phylogenetic tree of the lchAB protein sequences derived from Bacillus and other representative species. A total of 500 bootstrap replicates were made, and bootstrap values are indicated at each node. [file Image_7.TIF]

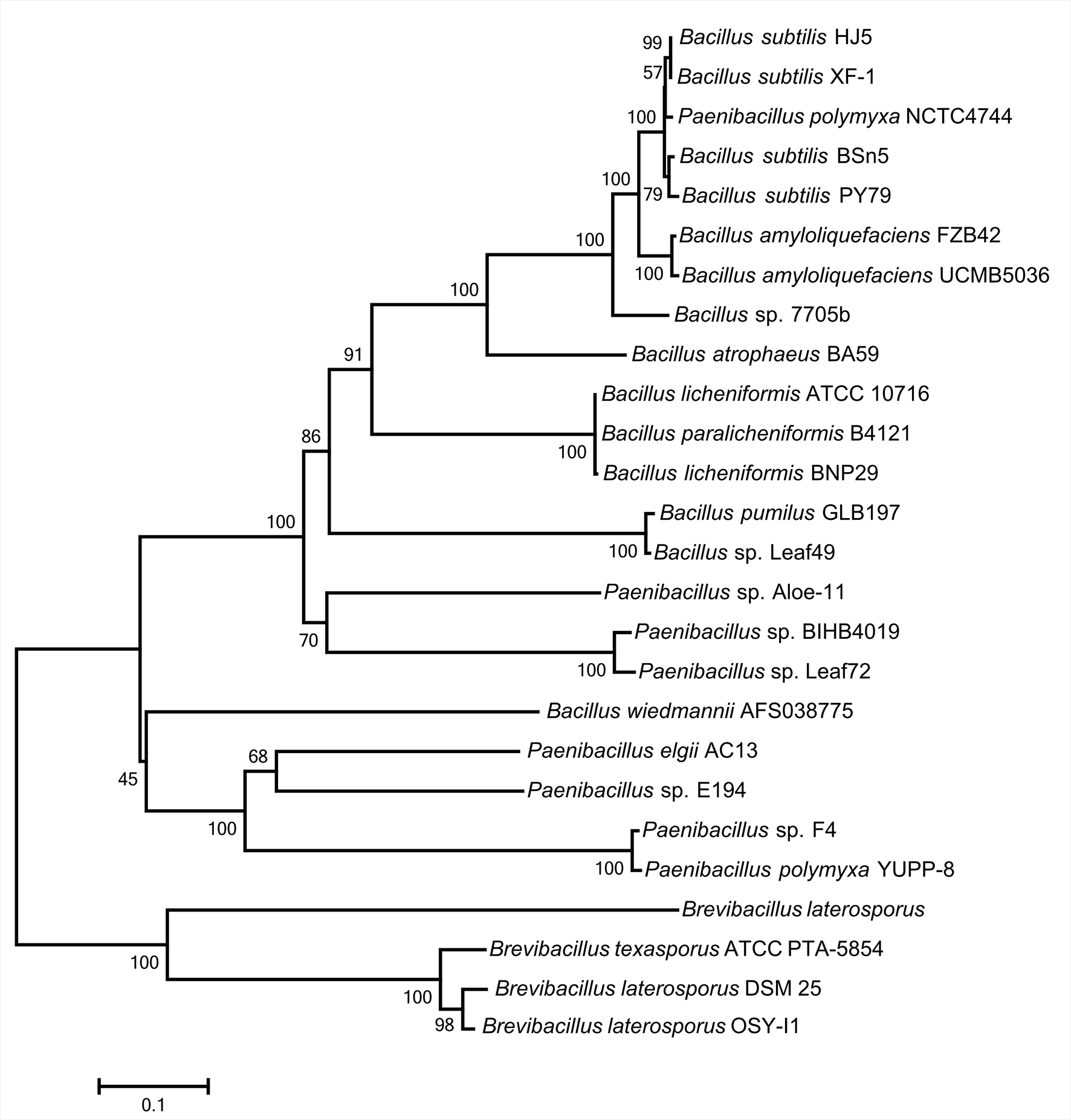

Supplement: Supplementary Figure 8 — Neighbor-joining phylogenetic tree of the lchAC protein sequences derived from Bacillus and other representative species. A total of 500 bootstrap replicates were made, and bootstrap values are indicated at each node. [file Image_8.TIF]

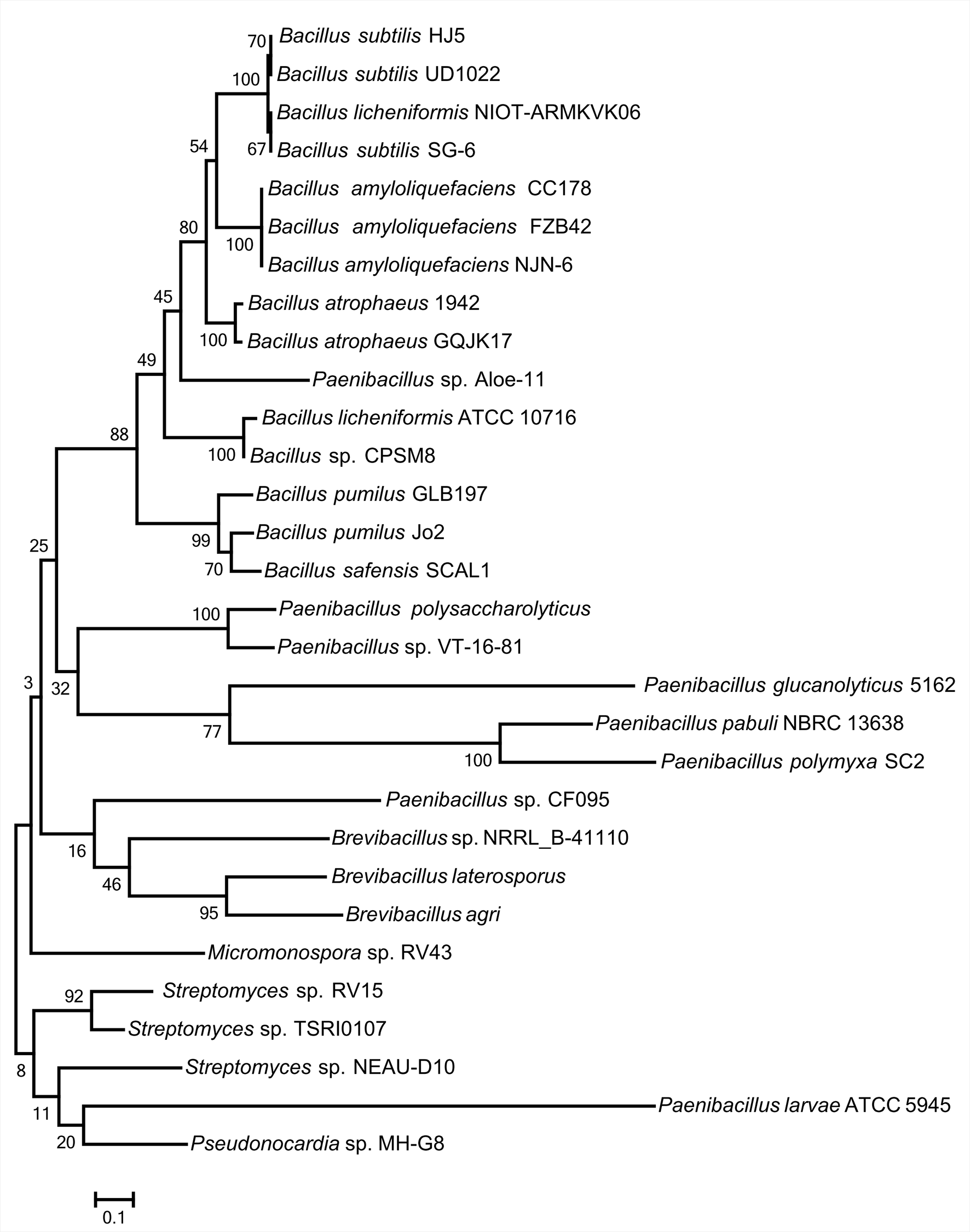

Supplement: Supplementary Figure 9 — Neighbor-joining phylogenetic tree of the lchAD protein sequences derived from Bacillus and other representative species. A total of 500 bootstrap replicates were made, and bootstrap values are indicated at each node. [file Image_9.TIF]

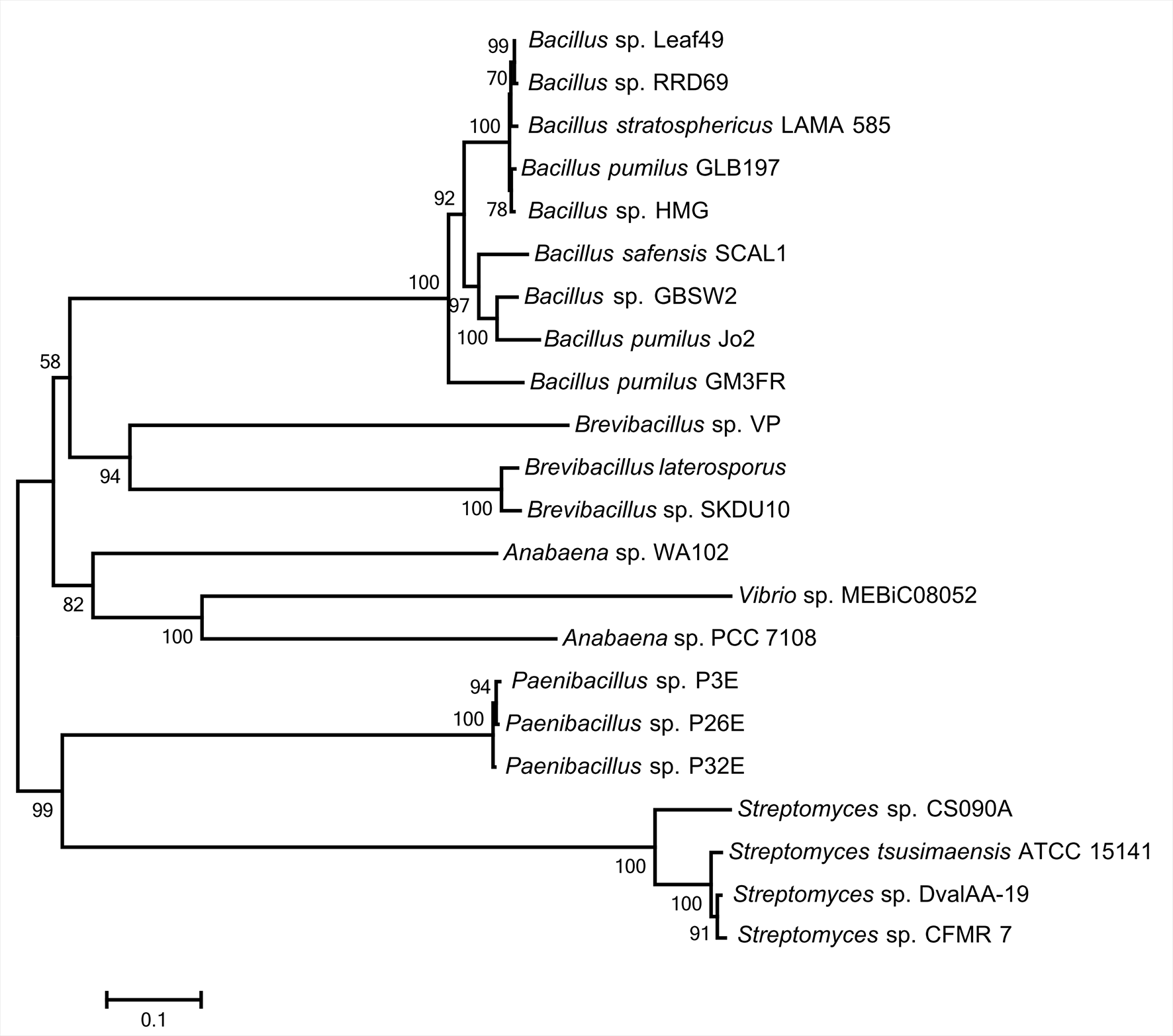

Supplement: Supplementary Figure 10 — Neighbor joining phylogenetic tree of the lgrB protein sequences derived from Bacillus and other representative species. A total of 500 bootstrap replicates were made, and bootstrap values are indicated at each node. [file Image_10.TIF]

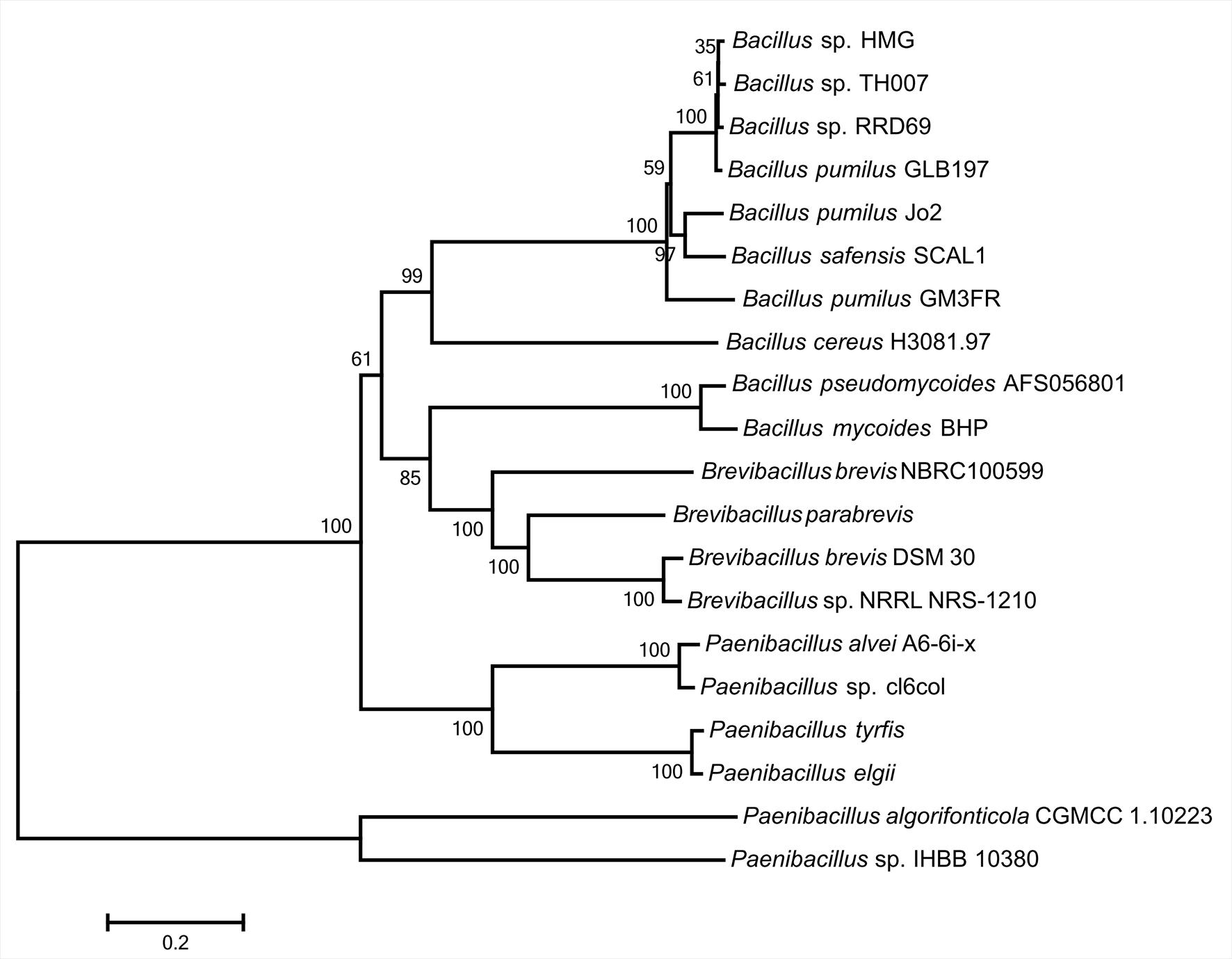

Supplement: Supplementary Figure 11 — Neighbor joining phylogenetic tree of the lgrD protein sequences derived from Bacillus and other representative species. A total of 500 bootstrap replicates were made, and bootstrap values are indicated at each node. [file Image_11.TIF]
